# Population genomics of resource exploitation: insights from gene expression profiles of two *Daphnia* ecotypes fed alternate resources

**DOI:** 10.1002/ece3.30

**Published:** 2012-02

**Authors:** Jeffry L Dudycha, Christopher S Brandon, Kevin C Deitz

**Affiliations:** 1 Department of Biological Sciences, University of South CarolinaColumbia, SC 29208; 2 Present Address: Department of Entomology, Texas A&M UniversityCollege Station, TX 77843

**Keywords:** Divergence, phenotypic plasticity, resource assimilation, seston, transcriptome

## Abstract

Consumer–resource interactions are a central issue in evolutionary and community ecology because they play important roles in selection and population regulation. Most consumers encounter resource variation at multiple scales, and respond through phenotypic plasticity in the short term or evolutionary divergence in the long term. The key traits for these responses may influence resource acquisition, assimilation, and/or allocation. To identify relevant candidate genes, we experimentally assayed genome-wide gene expression in pond and lake *Daphnia* ecotypes exposed to alternate resource environments. One was a simple, high-quality laboratory diet, *Ankistrodesmus falcatus*. The other was the complex natural seston from a large lake. In temporary ponds, *Daphnia* generally experience high-quality, abundant resources, whereas lakes provide low-quality, seasonally shifting resources that are chronically limiting. For both ecotypes, we used replicate clones drawn from a number of separate populations. Fourteen genes were differentially regulated with respect to resources, including genes involved in gut processes, resource allocation, and activities with no obvious connection to resource exploitation. Three genes were differentially regulated in both ecotypes; the others may play a role in ecological divergence. Genes clearly linked to gut processes include two peritrophic matrix proteins, a Niemann–Pick type C2 gene, and a chymotrypsin. A pancreatic lipase, an epoxide hydrolase, a neuroparsin, and an UDP-dependent glucuronyltransferase are potentially involved in resource allocation through effects on energy processing and storage or hormone pathways. We performed quantitative rt-PCR for eight genes in independent samples of three clones of each of the two ecotypes. Though these largely confirmed observed differential regulation, some genes’ expression was highly variable among clones. Our results demonstrate the value of matching the level of biological replication in genome-wide assays to the question, as it gave us insight into ecotype-level responses at ecological and evolutionary scales despite substantial variation within ecotypes.

## Introduction

The consumer–resource interaction is at the heart of community ecology and plays an important role in understanding the evolution of biodiversity. Many consumers exploit a range of resources, potentially requiring different mechanisms to use alternate resources effectively. Consumers face variation in space and time in the types of resources that are available, and this variation may lead to the evolution of plasticity or divergence into resource specialists. Ecologists have expended considerable effort to understand the ecological mechanisms governing plasticity and divergence in consumer–resource relationships, but relatively little information is available that identifies the genetic pathways that underpin such relationships. It is in these pathways that we may gain insight into the physiological, cellular, and molecular mechanisms that govern plasticity or are the targets of selection in evolutionary divergence of resource exploitation capabilities.

If consumers’ resource processing mechanisms are sufficiently plastic to accommodate a variety of resources, their performance at the organismal level will be relatively robust in the face of alternate resources. In contrast, a consumer whose resource processing is fixed may frequently find its performance constrained by a suboptimal diet. Evolutionary ecologists interested in the consequences of resource variation understandably have focused on fitness-based measures of performance (e.g., Fox 1993; Reznick and Yang 1993; Cruz–Rivera and Hay 2000; Tessier and Woodruff 2002). However, it is possible that different genotypes can achieve similar fitness responses via different mechanisms of acquiring and processing resources. This is a form of phenotypic plasticity, but may go unnoticed if it does not involve significant morphological or behavioral changes.

For some systems and interactions, ecological geneticists have been able to make significant progress in identifying and understanding genetic pathways that govern an ecological interaction because they involve simple relationships between genetic and phenotypic variation and between phenotypic variants and the interaction in question (e.g., Brunetti et al. 2001; Abzhanov et al. 2004; Rosenblum et al. 2004; Rosenblum 2006; Streisfeld and Rausher 2009). This is not the norm. Most species’ interactions with the environment involve a complex suite of traits, each of which in turn may be influenced by a broad set of genes. Thus, for most interactions, simply identifying genes that are important to the interactions represents a major challenge. Fortunately, as genomic approaches extend beyond traditional genetic models, it becomes possible to use them to identify key genes in complex interactions (e.g., Decaestecker et al. 2011).

**Figure 1:**
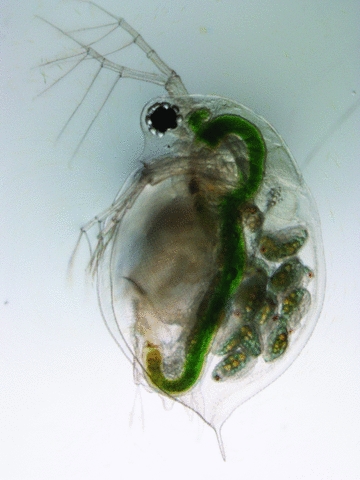
*Daphnia pulex*. Photo by Christine Ansell.

We sought to identify candidate genes that may play important roles in resource exploitation and its evolutionary divergence by capitalizing on the development of genomic tools in the ecological model consumer *Daphnia* (Fig. 1; Shaw et al. 2007; Eads et al. 2007; Colbourne et al. 2011; Jeyasingh et al. 2011). *Daphnia* are freshwater microcrustaceans that are often the dominant primary consumer in lakes and ponds (Hutchinson 1967). Though typically described as herbivores, because phytoplankton makes up the major part of their diet, *Daphnia* are actually omnivorous filter-feeders, ingesting, and potentially digesting, any suspended particles (seston) in the water. Thus, their diets can include bacteria, protozoans, fungi, and detritus in addition to the stereotypical phytoplankton (Lampert 1987).

*Daphnia* experience extensive temporal and spatial resource variation in nature. Seasonal variation in the composition of lake phytoplankton is perhaps most well documented (Sommer et al. 1986), but *Daphnia* also experience diel variation. Diel variation is influenced by both changes in the composition of the seston, and by the behavior of *Daphnia*, when they migrate between the epilimnion and hypolimnion (Leibold 1991; Winder et al. 2004; Reichwaldt and Abrusan 2007). At the metapopulation scale, any species of *Daphnia* will also experience substantial lake-to-lake variation in resource composition and availability. Thus, there is ample opportunity for evolution in response to variation. The genus has diversified into species that can be broadly assigned to water body type and season, and, consequently, have different general resource environments, but most species persists across some range of resource environments (Benzie 2005). We therefore can expect to find both diverged and plastic responses to resources.

We focused our efforts on identifying genes whose expression is regulated by resources at the mRNA level, and thus are candidates for pathways involved in plastic responses to resource variation. Differential gene expression in response to environmental variation is itself phenotypic plasticity, because gene expression is a phenotype. Identifying genes with plastic expression provides guidance on what molecular mechanisms may underpin organismal responses to resource variation by indicating cellular and physiological processes that potentially contribute to resource acquisition, assimilation, and allocation. Once candidate genes are identified in this manner, we can pursue functional studies at the molecular, cellular, and physiological levels.

In addition to identifying candidate genes with plastic expression, we sought to determine whether regulatory responses of these genes had diverged between *Daphnia* ecotypes associated with alternate habitats that present distinct resource environments. Populations of *Daphnia pulex* are typically found in temporary, forested ponds, where resource quality and quantity is high throughout most of the active season (Tessier and Woodruff 2002; Caceres et al. 2008). In contrast, *D. pulicaria* are typically found in large, permanent lakes, where year-round zooplankton populations drive phytoplankton communities to be dominated by grazer-resistant forms (Hu and Tessier 1995). In lake systems, *D. pulicaria* have scarce, poor-quality resources most of the year (Hu and Tessier 1995; Tessier et al. 2000). These putative species are closely related, hybridize frequently in the wild, and will produce fertile hybrid offspring in the lab (Pfrender et al. 2000; Dudycha 2004; Heier and Dudycha 2009). Some authorities recognize them only as subspecific variants (Dodson et al. 2009). Here, we refer to them as pond and lake ecotypes, since they occupy distinct habitats in the wild and have divergent traits associated with their habitats (Deng 1997; Pfrender et al. 2000; Dudycha 2001, 2003, 2004).

Tools developed by the *Daphnia* Genomics Consortium made it possible for us to survey gene expression for ∼6,000 genes simultaneously (Jeyasingh et al. 2011) and conduct a simple experiment where we manipulated resources available to the two ecotypes of *Daphnia* and observe the response of the gene expression profile (i.e., the transcriptome). Importantly, we constructed our experiment with replication that allows us to make inferences about an ecotype. To date, most experimental transcriptomics have focused on a single strain or genotype of the organism, and thus, the results are specific to the vagaries of that strain. By designing our experiment from the perspective of ecologists, our results are more likely to be relevant to how plasticity and divergence are occurring in wild populations.

## Materials and Methods

### Clones

*Daphnia* were isolated from lakes and ponds in southwestern Michigan (Kalamazoo and Barry counties) in the spring of 2008, brought into the lab, and allowed to establish clonal lineages under standard lab conditions. *Daphnia* from ponds were confirmed as *D. pulex* and those from lakes as *D. pulicaria* with the diagnostic allozyme LDH (lactate dehydrogenase; Hebert et al. 1989). Eight replicate clones of each ecotype were used. Each clone of the lake ecotype originated in a unique lake. Clones for the pond ecotype were obtained from six separate ponds (two ponds were each represented by two clones).

### Experimental exposures

Mothers of experimental individuals were isolated from stock cultures maintained at 10°C on a 12:12 L:D photoperiod and fed vitamin-enriched *Ankistrodesmus falcatus* (Goulden et al. 1982). Our culture medium was filtered (to 1 µm) lake water from Lake Murray, a large reservoir in central South Carolina, USA. To prevent surface film entrapment, each beaker of animals was dusted with a small amount of cetyl alcohol (Desmarais 1997). For each experimental clone, nine mothers were acclimated to 20°C for 1 week, where they were fed daily and transferred to fresh lake water every 2 days. At the start of the experiment, mothers were transferred to new beakers. The next morning, neonates for a particular clone were collected and pooled into a single beaker, giving us a set of experimental animals that had all been born in an 18-h window. From there, neonates were randomly distributed to new beakers at a density of five neonates per beaker containing 200-mL filtered lake water. For most clones, we had 50 experimental individuals, but mothers of a few clones produced slightly fewer neonates.

Beakers of experimental animals were randomly distributed within an environmental chamber at 20°C on a 12:12 L:D cycle. Experimental animals were fed a satiating level (at least 20,000 cells/mL) of the standard lab diet, vitamin-enriched *A. falcatus*, daily. *Ankistrodesmus falcatus* was quantified via cell counts on a hemocytometer, and on any day each beaker was provided the same quantity of food, though there was some day-to-day variation. Animals were transferred to new beakers thrice a week until the experimental animals were 10 days old.

We collected epilimnetic lake water from Lake Murray, brought it back to the lab and screened it through an 80-µm mesh to remove any zooplankton. This lakewater therefore contained the seston that forms the natural resource environment for zooplankton. We conducted a 24-h pulse experiment where half of the individuals, selected at random, were transferred into a beaker containing screened lakewater from Lake Murray; these were not fed *A. falcatus* that day. The remaining beakers were transferred to filtered water from Lake Murray and fed *A. falcatus* at a density of 23,000 cells/mL. Thus, the only difference between the Lake Murray seston treatment and the *A. falcatus* control was natural seston versus lab food.

After 24 h, eight randomly selected individuals from each clone were placed a single microcentrifuge tube, all water was removed with a pipettor, and then flash-frozen in liquid N_2_. Thus, for each clone in each resource environment, we obtained two independent tubes with eight individuals, and a third tube with the remainder. Once all animals were frozen, they were stored in a –80°C freezer.

### Characterizing resource environments

We characterized differences between the lab and field environments by measuring chlorophyll-*a* and by conducting a *Daphnia* growth rate bioassay. Three replicate samples of the unfiltered water from Lake Murray and of the *A. falcatus* suspension were taken on the day of experimental exposure and processed via standard methods for chlorophyll-*a* measurement. For each sample, 25 mL were filtered through a premuffled 25-mm GF/F glass fiber filter, dark-extracted in 7.0-mL acetone, and absorbance was measured in a fluorometer. The natural seston of Lake Murray contained 2.35 ± 0.06 SE ug chl-*a*/mL. Chlorophyll-*a* was about two orders of magnitude higher in our lab food, with 207 ± 2.00 SE ug chl-*a*/mL provided on the day of the experiment.

Despite the major differences in chlorophyll, the bioassay showed a smaller performance difference of *Daphnia* between environments. Our growth rate bioassay followed established methods for measuring juvenile specific growth rate, *g* (Lampert and Trubetskova 1996; Tessier et al. 2000), in which we estimate mass on neonates (*n* = 20) and 4-day-old juveniles (*n* = 20 per resource environment). We used the Geedey clone, a *D. pulex–pulicaria* hybrid clone that has been established as highly sensitive to variation in food quality (Tessier et al. 2000) and therefore is useful for measuring variation in resources from the perspective of the *Daphnia* themselves. In our bioassay, we found that *g* was 0.501 in the Lake Murray seston, and 0.592, approximately a 20% better performance, when fed *A. falcatus* at a concentration of 20,000 cells/mL. This shows that the Lake Murray seston was poorer as a food environment than the lab environment, but the low Chl-*a* level suggests that the Lake Murray seston provides an edible food other than green algae.

### Molecular methods

To ascertain transcriptome profiles, total RNA was extracted from one of the tubes of frozen tissue using a Qiagen RNeasy kit (Qiagen; Gerrmantown, MD, USA) with the optional on-column DNase treatment. After total RNA quality was checked on an Agilent 2100 Bioanalyzer (Agilent Technologies; Santa Clara, CA, USA) using RNA 6000 Nano chips, 1.5 µg of each sample was amplified and labeled using the Amino Allyl MessageAmp II aRNA Amplification kit (Ambion; Austin, TX, USA) according to the manufacturer recommendations. A total of 8 µg of amplified RNA was labeled with either Alexa Fluor 555 or Alexa Fluor 647 dyes (Invitrogen; Carlsbad, CA, USA). One microgram of each of the two-paired labeled samples was hybridized at 42°C during 16 h to third-generation *Daphnia* microarrays printed at the Center for Genomics and Bioinformatics at Indiana University. We used MAUI SC mixers, a 4-bay MAUI Hybridization system, and Pronto Universal Microarray Hybridization kits (Corning; Corning, NY, USA) according to the manufacturer recommendations. Array washes after hybridizations were performed using the Pronto kit. Subsequently, slides were scanned on a Perkin Elmer ProScanArray Express HT scanner (Perkin Elmer; Waltham, MA, USA) and the images quantified using Perkin Elmer ScanArray Express SP3 software (Perkin Elmer; Waltham, MA, USA).

We used two-color competitive hybridizations, where samples from the *A. falcatus* and Lake Murray treatments of a specific replicate clone were labeled with alternate dyes and cohybridized to the same microarray. To account for potential dye effects, dyes were reversed across replicates, such that half of the replicates of each ecotype had the *A. falcatus* treatment labeled with AF 555 and the Lake Murray seston-treatment labeled with AF 647, and half the replicates had the opposite labels.

The third generation *Daphnia* microarrays are 70-mer oligo arrays and are described in detail elsewhere (Jeyasingh et al. 2011; platform GPL13280 in the GEO database). These arrays include probes for roughly 6,000 unique genes, approximately 20% of the genes in *Daphnia* (Colbourne et al. 2011). The arrays include actin positive controls, and buffer-only and non-*Daphnia* negative controls in each of the 48 subarrays. Raw data from this experiment are publicly available in the GEO database under accession GSE31001.

We tested our microarray results using quantitative real-time polymerase chain reactions (qPCR) performed on eight differentially regulated genes. Five genes were chosen based on the known resource exploitation functions of homologous genes in other organisms; the remaining three were chosen so that all possible regulation patterns (i.e., up or down regulation in both ecotypes, up regulation in one ecotype but not the other, etc.) were represented. All genes were normalized to the reference gene *actin*, which showed zero differential expression in our microarrays (see below).

Because assaying all of the genes in all of the replicate clones would cost more than repeating the entire transcriptome analysis, we randomly chose three replicate genotypes of each ecotype for use in our qPCR assays. We used three different clones, rather than the single sample in standard microarray validations, to investigate the possibility for expression variation within ecotypes. Tissue for qPCR came from individuals that were independent from the samples used for the microarrays (i.e., a duplicate tube of frozen tissue generated by the experiment). Total RNA was extracted as for the microarrays, and purified RNA quality was determined on a NanoDrop-2000c spectrometer (Thermo Scientific; Waltham, MA, USA). We excluded samples with 260:280 ratios below 1.9, obtaining a new extraction from an independent sample when necessary. cDNA was synthesized by reverse transcription of 2 µg of purified RNA with random primers and 6-mM MgCl_2_ using a Promega GoScript kit (Promega; Madison, WI, USA).

qPCR primers were designed using Primer Express software (Applied Biosystems; Foster City, CA, USA) from gene sequences available through the *Daphnia* Genomics Consortium (http://wfleabase.org; see Supporting Information for primer sequences). Primers were blasted to the *Daphnia* genome to ensure that they would amplify only one fragment, particularly avoiding conserved regions of gene families. Individual primers did not necessarily span intron–exon boundaries and thus required measures to prevent genomic DNA amplification, including the DNAse treatment during extraction. Additionally, cDNA synthesis was performed without a reverse transcriptase enzyme and the resulting product was used in a qPCR assay to ensure carry-over genomic DNA was not responsible for observed expression level measurements.

We ran qPCR on an ABI Step One Real-Time PCR System (Applied Biosystems; Foster City, CA, USA). For each gene, five-fold dilution series of a standard *Daphnia* cDNA product were used to determine optimum primer concentrations by identifying the concentration that produced the qPCR efficiency nearest 100% (Table S1). PCR efficiencies were confirmed by rerunning the five-fold dilution series on the standard *Daphnia* cDNA product when experimental samples were assayed. PCR was performed for 40 cycles using the default Step One cycling parameters with an annealing temperature of 60°C for 1 min and denaturation at 95°C for 15 sec, preceded by initial enzyme activation at 95°C for 10 min. To ensure that only the intended fragment was amplified, qPCR products of each gene were run on an agarose gel, and each one produced a single band of the appropriate length. During each experimental qPCR, melt curve analysis confirmed that only a single fragment amplified. We used 4 µl (1 ng/µl) of cDNA product for most PCR reactions. This concentration failed to produce reliable Ct values for the gene PTM2, so we used 4 µl at 10 ng/µl for that gene. Reactions were run in triplicate and relative mRNA levels were calculated by the ΔΔCt method (Pfaffl 2001) using gene-specific primer efficiencies (given in Table S1).

### Data analysis

Microarray data were analyzed with the limma package of R (Smyth 2005). All arrays were assayed for data quality, and normalized using print-tip loess without background correction because nearly all spot-background correlations were below 0.2 (repeating analyses with background correction yielded identical results). An examination of the fluorescence distributions on each slide indicated that between-slide normalization would be useful, and this was done with the *Aquantile* method. From here, we fit a linear model (with *lmFit*) to the data and tested for significant differences in regulation using *global fdr* (global false discovery rate) to control for multiple comparisons. Our analysis used each clone as an independent replicate, and thus, statistically inferred differences in gene expression apply to the ecotypes as wholes. Restricting the analysis to include only six pond clones from independent ponds did not change the results.

For each probe that was identified as differentially regulated, we annotated the corresponding gene in the *D. pulex* genome using tools provided through the Joint Genome Institute (JGI) interface. First, we curate the structure of the gene model as predicted by JGI and other gene model prediction algorithms. This entailed verifying start and stop codons, intron–exon junctions, and the absence of premature stop codons. We also examined gene expression data in the *Daphnia* Genomics Consortium's web portal (http://wfleabase.org; Colbourne et al. 2005) to confirm that the sequence in question was an actively expressed region. The availability of tiling array expression data allowed us to confirm expression of each individual intron. Once the best gene model was identified, we used blastp on the *Daphnia* genome (assembly 1.1) to determine whether any paralogs could be found. When necessary, we identified similar genes to properly assign the *Daphnia* gene within a gene family. We also confirmed that the microarray probe sequences matched only a single locus in the genome.

To identify possible functions of the regulated genes, we identified the homologous genes in the *Drosophila melanogaster* genome and in the entire NCBI database. Function of the *Drosophila* homolog was determined from FlyBase (http://www.flybase.org; Tweedie et al. 2009) or literature when possible, and supplemented with NCBI's Conserved Domain Database (Marchler-Bauer et al. 2009, 2011).

We sought to determine if actin was differentially expressed in our environments, and thus assess its effectiveness as a reference gene in qPCR. To do so, we analyzed the *M*-values of the 48 actin spots that are distributed across each array (one in each subarray). First, we switched the sign on *M*-values where the lab resource sample was labeled in red so that all data were in the form of expression in the Lake Murray samples relative to the lab food samples. A linear model showed a significant effect of dye-direction, with samples where cDNA derived from animals fed the lab resource were dyed in green showed higher *M* values (*t* = 2.7264; *df* = 14; *P* = 0.0164), and allowed us to estimate the least squares mean for each dye direction. The green-dyed lab resource had an average *M* of 0.1208 ± 0.3712 SD, and the red-dyed lab resource had an average *M* of –0.2407 ± 0.4324 S.D. Averaging over the adjusted means of the 16 clones, the mean *actin M*-value was 0.0000 ± 0.064 SE and not significantly different from zero (*t* = 0.0004, *df* = 15, *P* = 0.999) suggesting that there was no general difference in expression of actin between treatments and that differences among clones are slight. There was also no significant difference from zero when we considered ecotypes separately (lake: *t* = 0.1540, *df* = 7, *P* = 0.882; pond: *t* = –0.3469, *df* = 7, *P* = 0.739). Therefore, it is reasonable to use actin as a reference gene to estimate relative gene expression of other genes between our treatments.

In the qPCR assays, we estimated the mean change in expression between the lab and Lake Murray resource environments for each clone by permuting the ΔΔCt equation (*n* = 2000) while shuffling the triplicate values. We then took the relative expression value from the mean of the permutations (Ritz and Spiess 2008). To test whether regulatory differences were significantly different from zero, we performed a second simultaneous permutation that randomly allocated replicate values between sample and control groups. Permutation analysis was performed using the *qpcR* package in R (Ritz and Spiess 2008).

## Results

**Figure 2:**
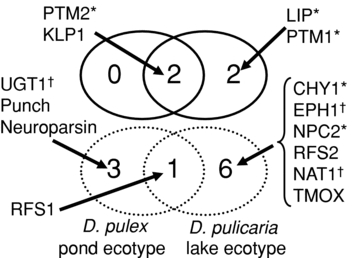
Differentially regulated genes in pond-adapted and lake-adapted ecotypes of *Daphnia*, including four genes with higher expression (solid-line ovals) and 10 with lower expression (dotted-line ovals) when *Daphnia* were fed seston from Lake Murray, SC relative to being fed a lab-reared algae *Ankistrodesmus falcatus*. Asterisks indicate genes associated with gut processes, daggers indicate genes associated with resource storage/allocation processes. See text for abbreviations and functional details.

Analysis of microarray data identified 14 genes that were differentially regulated in *Daphnia* given a 1-day exposure to alternate resource environments (Fig. 2), with several differences between the ecotypes. One gene was downregulated in the natural seston in both ecotypes, and two genes were upregulated in both ecotypes. In the pond ecotype, an additional three genes were downregulated in the natural seston, and none were upregulated. In the lake ecotype, six additional genes were downregulated in the natural seston, and two additional genes were upregulated. Curation and identification of homologs showed that many of these differentially regulated genes have a clear functional relationship to resource processing (Tables 1 and 2; detailed in Supporting Information). Some additional genes have a plausible role in resource exploitation, and others have no homology to genes with known function.

**Table 1:** Gene symbols, microarray probe identifiers, and expression levels in the microarray experiment for genes that differed significantly between treatments in at least one ecotype. *A* is the average fluorescence level across treatments. *A*-values for spotting-buffer controls were ∼ 4.1. *M* is the relative expression of the field seston-treated individuals to the controls, in log_2_ units. Boldface type indicates significantly different expression between the field seston treatment and the lab food control

		Pond ecotype		Lake ecotype	
			
Gene	Probe ID	*A*	*M*	*A*	*M*
PTM1	Dp001627	6.83	1.78	7.38	**2.31**
PTM2	Dp008924	6.15	**1.87**	6.83	**2.07**
CHY1	Dp003106	7.81	0.06	7.56	**–0.90**
NPC2	Dp003611	12.75	–1.82	14.09	**–1.58**
LIP	Dp001242	8.56	0.36	9.21	**1.07**
EPH1	Dp003130	8.87	–0.67	9.73	**–1.01**
UGT1	Dp004865	7.15	**–1.45**	7.69	–0.99
Neuroparsin	Dp009109	7.10	**–1.41**	7.87	–1.06
KLP1	Dp001037	6.07	**2.06**	7.22	**1.70**
Punch	Dp007040	6.38	**–0.83**	6.92	–0.25
NAT1	Dp005397	6.67	–0.77	8.01	**–1.10**
tMOX	Dp006883	10.03	–1.29	12.01	**–1.47**
RFS2	Dp004320	8.99	–0.81	9.93	**–1.07**
RFS1	Dp000454	8.35	**–1.24**	9.04	**–1.60**

**Table 2:** Homologs of differentially regulated genes inferred from blastp. Best matches are given for *Drosophila melanogaster*, and the best match in any organism

Gene	*Drosophila* locus	e-value	Other organism	Locus in other organism	e-value
PTM1	Obstructor E	2.00E−04	*Tribolium castaneum*	Peritrophic Matrix Protein 3	7.00E−08
PTM2	Obstructor E	7.00E−07	*Nematostella vectensis*	predicted protein	5.00E−08
CHY1	Jon65Aiv	7.00E−38	*Caligus rogercresseyi*	Chymotrypsin-C precursor	3.00E−40
NPC2	Niemann-Pick type C−2a	3.00E−18	*Litopennaeus vannemei*	ecdysteroid-regulated protein	5.00E−20
LIP	CG5966	2.00E−55	*Acyrthosiphon pisum*	similar to triacylglycerol lipase, pancreatic	8.00E−119
EPH1	jheh3	1.00E−02	*Monodelphis domestica*	similar to abhydrolase domain containing 7	1.00E−64
UGT1	CG30438	3.00E−67	*Tribolium castaneum*	similar to CG30438	3.00E−77
Neuroparsin	dumpy	2.80E−01	*Rhodnius prolixus*	Neuroparsin 1 precursor	4.00E−15
KLP1	Tm2	1.30E−02	*Mus musculus*	centromere-associated protein E	5.00E+00
Punch	Punch	2.00E−79	*Saccoglossus kowalevskii*	GTP cyclohydrolase	7.00E−81
NAT1	CG5783	2.00E−09	*T. castaneum*	similar to CG5783	2.00E−12
tMOX	olf314	8.90E−02	*Saccoglossus kowalevskii*	similar to CG5235	1.00E−03
RFS2	kuzbanian	5.70E+00	*Myxococcus xanthus*	hypothetical protein	5.00E−17
RFS1	none	N/A	*Saccoglossus kowalevskii*	XP_002734638	2.00E−03

### Gene curation and functional comparisons

We identified apparent homologs of the regulated genes in other organisms using public databases. We specifically searched for homologs in *D. melanogaster*, the arthropod most likely to have functionally characterized genes. Homologs of several regulated genes clearly have a function related to resource assimilation or allocation, suggesting that our approach identifies relevant candidate genes even though the total number of significantly regulated genes is low compared to single-strain transcriptome studies.

Four genes have apparent functions directly related to digestion and gut processes. Peritrophic matrix proteins 1 (PTM1, upregulated in the lake ecotype) and 2 (PTM2, upregulated in both ecotypes) are structural constituents of the petritrophic matrix, the inner lining of the gut that is sloughed off as food passes through the gut. Chymotrypsin (CHY1, downregulated in the lake ecotype) is a member of the trypsin family of digestive enzymes, a group of serine-type endopeptidases involved in proteolysis. The best match in *Drosophila* is a member of the Jonah class of genes, which are expressed exclusively in the midgut (Carlson and Hogness 1985a, b). Niemann–Pick Type C2 (NPC2, downregulated in the lake ecotype) is an apparent homolog of a human gene that, when mutated, results in Niemann–Pick type C2 disease, a condition that results from accumulation of low-density lipoprotein cholesterol in lysosomes. However, in *Drosophila*, the gene (*Npc2a*) is involved in ecdysteroid pathways, and is highly expressed in midgut and salivary gland tissue (Huang et al. 2007). Mutations result in ultrastructural defects of Malpighian tubules (Huang et al. 2007), which are distally blind branching structures extending from the mid-/hindgut interface, where they are involved in excretion and osmoregulation. Though described in insects, myriapods, and arachnids, we are unaware of any reports of Malpighian tubules in crustaceans, and this result suggests further anatomical investigation of crustacean guts is warranted.

Four genes have homologs with functions that may be related to resource assimilation or allocation, but are not necessarily associated with the gut. Lipase (LIP, upregulated in the lake ecotype), is a triacylglyceral lipase that has no known function in the *D. melanogaster* homolog (CG5966), but belongs to the pancreatic family of lipases and is associated with lipid metabolism in mammals and birds. Epoxide hydrolase 1 (EPH1, downregulated in the lake ecotype) is also a member of the esterase/lipase family of genes, but is part of the juvenile hormone (JH) pathway. The JH pathway regulates life-history plasticity and is important in determining patterns of resource allocation. In *Drosophila* the best match is to *jheh3* (juvenile hormone epoxide hydrolase 3) and an unnamed gene (CG1882) that was identified from lipid droplets. Neuroparsin (previously curated and named by H. Dircksen in the JGI *D. pulex* genome database) is a protein produced by neurosecretory cells and has several activities including inhibition of JH and stimulation of reabsorption in the hindgut (among others). Therefore, it may play a role in resource allocation through its effect on life-history-regulating hormones, or in resource assimilation through its effect on gut processes. UDP-dependent glucuronyl transferase (UGT1, downregulated in the pond ecotype), may have a role in resource allocation since domains identified in the NCBI Conserved Domain Database indicate that it belongs to the glycosyltransferases, a group of genes involved in carbohydrate transport and metabolism. However, this family of genes has a wide range of biological functions, and thus, the potential link to resource allocation is weaker than for LIP, EPH1, and Neuroparsin.

Four genes have apparent homologs with known functions or functions that can be inferred from conserved domains, but have no obvious link to resource acquisition, assimilation, or allocation. Kinesin-like protein 1 (KLP1) was upregulated in both ecotypes, and appears to be a motor protein associated with centromeres. Punch (downregulated in the pond ecotype), a clear homolog to the *Drosophila* gene *punch*, is a GTP cyclohydrolase I, a group of enzymes that catalyzes the conversion of guanosine triphosphate (GTP) into dihydroneopterin triphosphate. Though involved in many functions in *Drosophila*, including the mitotic cell cycle, cuticle pigmentation, pteridine biosynthesis, embryonic patterning, compound eye pigmentation, and chitin-based cuticle development, none are obviously associated with resource processing. One possibility is that Punch may play a similar role to the peritrophic matrix proteins, which are also involved in chitin-based cuticle development. N-acetyltransferase 1 (NAT1, downregulated in the lake ecotype) is functionally annotated only based on conserved domains; no specific homologs have any functional information. N-acetyltransferases are involved in a wide range of functions, but none that have been described are connected to resource processing. A possibly truncated monooxygenase (tMOX, downregulated in the lake ecotype) is apparently a dopamine beta-monooxygenase that is suggested to be involved histidine catabolism. The JGI v1.1 genome sequence of *D. pulex* contains a premature stop codon, indicating a truncated, and possibly nonfunctional, polypeptide. However, expressed sequence tag sequences in wFleaBase do not support the premature stop, so this may reflect an error in JGI v1.1.

Finally, two genes that were differentially regulated could not be assigned any significant homology and contained no conserved domains. In the absence of any homologs or functional information, we named these genes Regulated by Field Seston 1 and 2 (RFS1 and RFS2), though they have no sequence homology with each other. RFS1 was downregulated in both ecotypes, and has very weak homologs (*E* = 0.01 or worse) in mammals, hemichordates, and *Caenorhabditis elegans*, but none had any associated functional information. RFS2 (downregulated in the lake ecotype) had a reasonable match to a hypothetical protein in *Myxococcus xanthus* (*E* = 5e–17), but the nearest match with any functional information was to the *D. melanogaster* gene *kuzbanian* (*E* = 5.7), a gene involved in phagocytosis in the innate immune response. Though this match is so weak that we would normally ignore it, matching residues were spread throughout the amino acid sequence, and it would be interesting if these matches turned out to be at functionally significant locations. However, no such information is available for *kuzbanian*.

### Consistency of expression differences across genotypes

**Figure 3:**
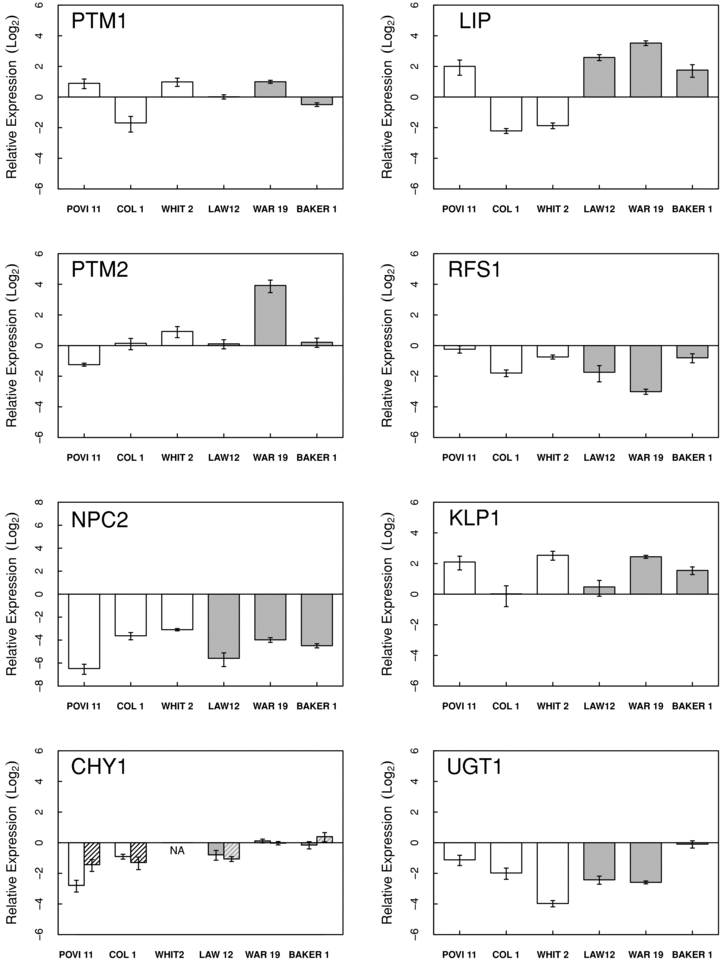
Relative gene expression of eight genes in *Daphnia* fed seston from Lake Murray, SC compared to being fed the lab-reared algae *Ankistrodesmus falcatus*. Relative expression is given in log_2_ units; +2 indicates a four-fold increase in expression, whereas –2 indicates a decrease to one-fourth. White bars show three pond-ecotype clones, gray bars show three lake-ecotype clones. Error bars are standard deviation. In all cases where error bars do not cross zero, expression differences were significantly different from zero (*P* < 0.001) in a permutation test. For the gene CHY1, bars are shown from two different pairs of primers (see text for explanation).

Our qPCR assays showed at least partial concurrence for each of the tested genes with the microarray results (Fig. 3), but highlighted that there may be substantial variation of expression responses within ecotypes. These differences were particularly notable when the microarray analysis showed no expression change for an ecotype. This is an important result because it shows that the common practice of pooling samples from multiple genotypes obliterates information that is potentially important for understanding evolutionary questions.

We chose to conduct qPCR with the five genes whose inferred functions were most clearly associated with resource assimilation (the four gut-associated genes, PTM1, PTM2, NPC2, and CHY1; and LIP; the triacylglyceral lipase). We chose three additional genes (RFS1, KLP1, UGT1) to include all of the regulatory patterns observed in the microarray analysis.

In the microarray comparison, LIP was upregulated in the lake ecotype but was not differentially regulated in the pond ecotype. Clone-specific qPCR confirmed that clones originating in lakes upregulated LIP, with substantial variation of the degree of upregulation (3–12 fold increase). Pond clones showed both down- and upregulation: WHIT 2 and COL 1 downregulated LIP, whereas POVI 11 upregulated LIP.

PTM1 and PTM2 were both upregulated in the microarray comparison of the lake ecotype, but only PTM2 was upregulated in the pond ecotype. However, qPCR showed that these were not consistently upregulated across the subset of genotypes we tested. Of the lake genotypes tested, only WAR 19 showed upregulation for the peritrophic matrix proteins. The 16-fold upregulation of expression in WAR 19 is in stark contrast to the absence of differential regulation in the other two lake clones. We found qualitative variation among the pond clones. Both genes were upregulated in the WHIT 2 clone, whereas PTM1 was downregulated in COL 1, and PTM2 was downregulated in the POVI 11 clone.

In the qPCR tests, NPC2 showed a large decrease in expression across all clones of both ecotypes, with relatively little variation among clones. This confirms the microarray result for the lake ecotype, and suggests that the absence of an effect in the pond ecotype may have been a false negative in the microarray analyses. This is unsurprising, since the methods for controlling for multiple comparisons inherent in transcriptome profiling focus on reducing false positives, not false negatives.

We had substantial difficulty designing suitable primers to test the expression of CHY1. In part, this was because it is part of a large family of genes, and we needed to ensure that our primers would match only the gene that contained the microarray probe. However, our initial primers, designed off of the *D. pulex* genome sequence, failed to amplify in our clones. We verified that these primers worked in SL14, a clone from Oregon, USA (the same geographic region as TCO, the clone whose genome was sequenced). Since our experimental clones are all from Michigan, it is possible that this reflects geographic sequence variation. We then examined EST sequences for CHY1 found in wFleaBase, which were derived from a clone other than TCO, and discovered that they differed substantially from the genome sequence. We designed primers off of the EST sequences, and these amplified fragments in most, but not all, of our experimental clones. We ran qPCR using two different pairs of these EST-derived primers and found that they yielded concordant results. These results contrasted with the microarray results. In the microarray, CHY1 was downregulated in the lake ecotype only, but in the qPCR tests, downregulation was seen in both pond genotypes in which amplification was successful, but in only one of three genotypes from lakes. Together, the apparent variation in sequence and regulation, coupled with expansion of the gene family in the *D. pulex* genome, indicate that these genes may be linked to current evolutionary change in resource processing.

The remaining genes (RFS1, KLP1, UGT) were chosen to cover all regulation patterns we observed in the microarray analysis. RFS1 was the only gene downregulated in both ecotypes according to the microarrays, and our qPCR results confirmed this in all clones tested. The microarrays showed that KLP1 was upregulated in both lake and pond ecotypes, which was confirmed by qPCR in five of the six genotypes. Though both RFS1 and KLP1 showed consistent regulatory patterns between ecotypes that were qualitatively confirmed in the clone-specific qPCR, we observed substantial quantitative variation in the qPCR tests. Finally, our qPCR tests showed that UGT1 was downregulated, but variably so, in both ecotypes. Microarray analysis identified UGT1 as being downregulated in the pond ecotype, but not the lake ecotype. As with NPC2, the difference may be due to a false negative in the microarray analysis.

## Discussion

Ecologists are increasingly challenged to understand the physiological, cellular, and molecular mechanisms by which organisms respond to variation in their environments because ecologically significant tradeoffs are resolved at these levels. In this work, we focus on identifying phenotypic plasticity of gene expression that may form the foundation for plasticity of traits more familiar to ecologists. In addition, an understanding of the molecular basis for responses to variation offers insight into evolutionary targets that may be the basis for adaptive divergence and the expansion of biodiversity. Genome-scale approaches have the potential to identify key genes for complex ecological responses that cannot reliably be approached via serendipity or candidate genes, but that potential is only beginning to be exploited.

We used a genome-wide analysis of expression in the ecological model organism *Daphnia* and identified 14 candidate genes whose expression was phenotypically plastic when fed alternate resources. Since our experiment was conducted to allow only the available resource to differ, we can conclude that these differentially regulated genes play some role in the response to resource variation, even though functional information at the molecular level is not available for this genus. However, we inferred that many of these genes have likely functions associated with gut processing, resource assimilation, or resource allocation based on apparent homology to genes in genetic model organisms or evaluation of conserved gene domains. This demonstrates that the genome-wide approach can usefully identify genes that are likely to play a role in complex ecological responses.

One of our goals was to identify candidate genes that may be relevant to the resource responses of an ecotype, rather than a single strain, since differences at the level of ecotype may be associated with ecological divergence. Most transcriptome profile analyses use some strategy to minimize the variation among replicate samples (e.g., by using a single strain or pooling RNA from a genetically heterogeneous population) in order to maximize the sensitivity to detect subtle changes in gene regulation. Historically, this was a sensible strategy because genomic techniques were developed by biologists interested in highly conserved molecular functions, where there is a commonplace assumption that what happens in *Drosophila* also occurs in humans. However, ecologists and evolutionary biologists are interested in traits that are likely to vary widely within and among populations, and tend to focus on taxonomic levels that represent ecologically significant genetic diversity. Thus, we believe that the focus on genetic variation minimization is often not especially useful in studies of evolutionary ecology. This meant turning to a population genomic level of investigation because we are not trying to make inferences that are limited to a specific pond or lake. Instead, we seek to identify genes that may reflect the important processes that distinguish pond populations in general from lake populations.

Eleven genes that we identified as resource regulated with the genome-wide approach were resource regulated in only one ecotype, and these may thus be genes whose responsiveness (or canalization) with respect to resources contributes to the evolutionary divergence of *Daphnia* into ponds and lakes. Though these may be targets of selection underlying the divergence between ecotypes, we cannot yet conclude this, because by conducting clone-specific tests of regulation, we were able to reveal variation within ecotypes. Additionally, we found two genes where the absence of a regulatory response for an ecotype in the microarrays may have been a false negative. We have identified genes that would be good subjects for detailed analysis of the metapopulation structure of genetic variation in expression to establish any association between gene expression variation and in situ resource variation. Such an analysis could potentially include selection gradient analysis to determine whether the gene expression phenotype is associated with differential fitness in alternate habitats.

Three genes showed similar regulatory patterns in both ecotypes, only one of which (PTM2) has a clear connection to resource exploitation. We interpret these genes as being part of the shared mechanisms by which *Daphnia* respond to resource variation generally.

Although our approach offers some insight into the mechanisms that may be involved in a consumer–resource interaction, two additional considerations are important. First, the inferred functions need to be determined via direct genetic assays. In many cases, a general function can be reliably determined via comparative analysis, but the potential for evolutionary expansion of gene families and sub- or neo- functionalization of new gene duplicates limits inference about specific functions of genes and, importantly, their expected regulatory responses to an environmental change. For example, we do not have the functional information about the peritrophic matrix proteins to know whether the direction of their regulation is as expected, what aspects of the resource environment are driving their regulation, or what other genes are linked to them in molecular pathways. One advantage of working with *Daphnia* is that it has become the subject of an extensive network of genetic researchers, and genetic tools are appearing that will allow us to determine the molecular function of genes we have identified in this study (Robinson et al. 2006; Tsuchiya et al. 2009; Kato et al. 2011; Colbourne et al. 2011).

Second, we recognize that we have only identified a subset of genes involved in responses to the resource variation that *Daphnia* experience. Other genes may be important in other resource contexts (e.g., resource quantity variation, seasonal variation of the seston, or algal taxa with different nutritional value) or at other developmental stages, and additional important genes may not have been represented on our microarrays. Therefore, our results should not be viewed as an attempt at identifying a comprehensive set of resource-responsive genes, but rather as an approach that identifies genes that are good candidates for research aimed at linking ecology, evolution, and functional genetics.

## Data Accessibility

All microarray data from this experiment are available in the GEO database under accessions GSE31001. Additional data (primer sequences, optimum primer concentrations, qPCR efficiencies, and gene curation and annotation information) are in the online Supporting Information.

## Supporting Information

**Table S1.** Primer sequences, product sizes, concentrations and efficiences for qPCR.
