# Supplementary material for: Population genomics of resource exploitation: insights from gene expression profiles of two Daphnia ecotypes fed alternate resources
Source: Ecol Evol. 2012 Feb;2(2):329–40. doi: 10.1002/ece3.30 (PMC3298946; doi:10.1002/ece3.30)
Supplement: Supplementary file 1 [file ece30002-0329-SD1.pdf]

Supplementary Information: qPCR specifications.

Supplementary Table 1. Primer sequences, product sizes, concentrations and efficiences for qPCR.

Gene	Forward primer (5'-3')	Reverse primer (5'-3')	Amplicon size (bp)
Actin	GATTGATGATGGGTTTTCCTTCTC	TGGCATGAAGATAGATTTGGGTATT	136
PTM1	AGCACATCACTACCTTTGGTTTTCT	GCAGTCGATCCTGGCAGACT	65
PTM2	GTCGTCGAGCGCTTATTTCG	AGAGCCCTTATTTCTTTCCTTAACTAGA	80
NPC2	GCGTGACGATAACGGCAAT	CTAGGCTACGTCCAATTACAGAAGTTT	73
CHY1 – pair #1	GAACTTGGCGTGGTGGTAGTAGA	ATCCGTACGGTTACACAAGACTCA	100
CHY1 – pair #2	GGTGGTGCACATGACTTTACTTG	TCCTGTACTCCGCGAAGTGA	100
LIP	TCGGTTGCATTGTCATCGA	TGATTGATTGGGCGATGGA	58
KLP1	TTGCTTCCTTGAGAGCATTGG	TCGAAACTGGGACATGAAGGT	65
RFS1	AATGGATTGAACCCCACAAAAA	ATGGTGACCACTACCGGAATG	70
UGT1	TGGGATTGCCGTTCATTAGC	CGCGGACCGCCTTGT	54

Gene	Optimum Primer Concentration (nM)	qPCR Efficiency (%)
Actin	100	96
PTM1	100	98
PTM2	200	96
NPC2	200	96
CHY1 – pair #1	200	96
CHY1 – pair #2	200	96
LIP	100	98
KLP1	100	101
RFS1	400	99
UGT1	75	97

Supplementary Information: Details of Gene Curation and Comparative Analysis (genes are given in alphabetical order)

Chymostrypsin – downregulated in lake ecotype
Summary:

The gene CHY1 (Chymotrypsin) is downregulated in *Daphnia pulicaria* (the lake ecotype) when they were provided seston from Lake Murray, SC as a food source compared to when they were provided the lab-reared food *Ankistrodesmus falcatus*. CHY1 is represented on the *Daphnia* 3rd generation microarrays by probe Dp003106, and is located at Scaffold 18:1129087-1130593. It was manually curated in the *Daphnia pulex* genome sequence based on the gene model predicted by the PASA algorithm, and supported by evidence of expressed sequence tags and tiling array expression in wFleaBase. Many paralogs were found in the *Daphnia* genome via tblastn; this appears to be part of a large family of genes. However, the blast match to the next best gene (scaffold 99) was 2e-43, compared to the self-match of e-138. Blasting the probe sequence to the *Daphnia* genome found partial matches on Scaffold 99 (two separate, but identical 33bp matches) and Scaffold 14 (a separate 40 bp match). It is therefore possible that our array expression results are influenced by these additional genes.

Blastp at NCBI show that this is clearly a trypsin, however the first ~100 residues are not part of any blast matches. Conserved domains in the gene include the cleavage site, active sites, and substrate binding sites of trypsins. Best hits are to Chymotrypsin-C precursor (*Caligus rogercresseyi*; E=3e-40), and a putative Chymotrypsin-BI precursor(*Pediculus humanus corporis*; E=6e-40). In *Drosophila melanogaster*, the best match is to Jonah65Aiv, part of a large family of serine-type endopepdidases involved in proteolysis that are expressed exclusively in the mid-gut.

Microarray probe sequence (Dp003106)
GTCGAGGGCCATGCAACGGCGATAATGGAGGGCCGTTGATTTTCAGAGAGTCAAATGGTCGATGGA
AACA

Peptide sequence:
```
>jgi|Dappu1|442949|dud_PASA_GEN_1800067
MAVEKFRSLFLTAFLIAAASGSIAPLKLDTQEVVLNLQPPSFSIKLNLRNDSSLLDLPWTNGKEQEKVPA
PTDTTISPIRTSSSRAPSTTPSYWYGDCGVANLMDEATDRIVGGVQAIPNEFPWQAFVKVETNAGNIYYC
GGSLIADRWILTSARCVLIPGQTLRYLTIYLGAHDITASYEANRRVYNGYEAYIHPEWNPSTQAGDIALI
KLCNIVTYTQYIRPVCLATYNEPSYVNSQVAVAGWGTTSDGSYTLSPVLREVTVPVISNTQCSNYYGSAV
VTSKVMCTTGMLSRGPCNARPLIFRESNGRWKQIGIVSFVSSQGCQSGYPYGYTRLSSYSTWVQNVMSSY
SGSPSTTTTPSPSSGSQTFAILSLVISLFLLMQL*
```

Epoxide hydrolase 1 – downregulated in lake ecotype
Summary:

The gene EPH1 (Epoxide hydrolase 1) is downregulated in *D. pulicaria* when they were provided seston from Lake Murray, SC as a food source compared to when they were provided the lab-reared food *Ankistrodesmus falcatus*. EPH1 is represented on the *Daphnia* 3rd generation microarrays by probe Dp003130, and is located at Scaffold 5:121627-126041. It was manually curated in the *Daphnia pulex* genome sequence based on the gene model predicted by the PASA algorithm, and supported by evidence of expressed sequence tags and tiling array expression in wFleaBase. Nine potential paralogs were found in the *Daphnia* genome via tblastn (each with an E-value of e-5 or better), several of which were automatically annotated as epoxide hydrolases. However, none of these were supported by EST sequences or tiling array expression data

suggesting that they are all nonfunctional. The sequence of probe Dp003130 blasts only to EPH1 on Scaffold 5.

The best blastp matches (E = 1e-64) were to predicted abhydrolase domain containing 7 genes in *Gallus gallus* and *Monodelphis domestica*. In *Drosophila melanogaster* there was a weak hit (E = 0.01) to juvenile hormone epoxide hydrolase 3, and a similar hit to CG1882, an unnamed gene whose protein product is found in lipid droplets. The best known gene matches are to the CEEH family in *Caenorhabditis* (CEEH = *Caenorhabditis* epoxide hydrolase). NCBI's conserved domain database showed that the gene is a member of the esterase/lipase superfamily, a group of enzymes that act on carboxylic esters, and contains the alpha/beta hydrolase fold found in epoxide hydrolases.

Microarray probe sequence (Dp003130):
AGCCAAATGGGCGGAAAACTTCACTCTTGGCCTGATTCCCGAAGCGTCACATTGGGTGCAACAAGA
CGCT

```
Peptide sequence:
>jgi|Dappu1|306474|PASA_GEN_0500143
MSSTRPNLAVRIAVQCVAWALSAGMTIVLLPFLILFWLWSALTDYSDTVKKKKKSTSKGSLVSRGLGASH
YVQLKDVSIHYIEAGDRSRPLMLCLHGFPEFWFSWRHQLKEFSTTHRVVAVDLRGYGDSDKPNGRDAYKM
DKLVDDVRQIIDILGNGKCDVLLAHDWGAGIGWELVIRHPELVGRFVPMNCPHPAAFIDIISTEYSQILK
SWYMIFFQLPVVPEKLLTAFGASLFCWVFQRPGLEHKDAKAYLDLYQHPSDLTGPINYYRSMIDPDTMGQ
PGKRVKVPTLLIWGNEDRFLNISMAHRSAKWAENFTLGLIPEASHWVQQDAPRSVNEKIREFL*
```

Kinesin-Like Protein 1 – Upregulated in both ecotypes

Summary:

The gene KLP1 (Kinesin-Like Protein 1) is upregulated in both *Daphnia pulex* and *D. pulicaria* when they were provided seston from Lake Murray, SC as a food source compared to when they were provided the lab-reared food *Ankistrodesmus falcatus*. KLP1 is represented on the *Daphnia* 3rd generation microarrays by probe Dp001037, and is located at Scaffold 44:624798-626128. It was manually curated in the *Daphnia pulex* genome sequence based on the gene model predicted by the Gnomon algorithm, and supported by evidence of expressed sequence tags and tiling array expression in wFleaBase. Blastp found two paralogs in the *Daphnia* genome, on Scaffolds 10 and 2 respectively.

The function of KLP1 appears to be a motor protein associated with centromeres, as KOG places it in the Kinesin superfamily, in particular subfamily KAR3 in family 10. However, blastp at NCBI identified no conserved domains. No blastp matches are particularly strong, but the best blastp matches (e=-05) are for centromere-associated protein E (a kinesin) in mouse, and for a hypothetical protein in body louse. Matches at e = -4 are to kinectins in *B. taurus* and Zebrafinch, and to the "GRIP and coiled-coil domain-containing protein, putative" in bodylouse. Other matches have indications that they are involved with chromosome segregation. After that, matches get gradually worse, but the terms myosin, tropomyosin, and muscle all appear frequently. The best match in *Drosophila* is to tropomyosin 2 (Tm2; E = 0.013), but blasting the *Drosophila* Tm2 protein sequence to *Daphnia* found many regions that were better matches to Tm2 than is KLP1.

This appears to be part of a family of 3 or 4 genes in *Daphnia.* The paralogs are on scaffolds 10 and 2. The Scaffold 10 gene (JGI_V11_98611) has been annotated by A. Schurko as SMC1 (structural maintenance of chromosome protein), and is supported by clear EST and tiling array expression data. The scaffold 2 gene(s) are uncurated, but appear to be duplicates

separated by about 60kb. The gene on scaffold 10 is represented by two microarray probes (Dp003689 and Dp007943), and the genes on scaffold 2 are both represented by Dp006947. The probe for KLP1 does not match any of these genes; therefore we conclude that the differentially expressed gene in our experiment really is KLP1, and not one of the related genes.

Microarray probe sequence (Dp001037):
CCGTTTTCACTGTTGGATTAGCTACCCCTGGTCTCATACACAACGAAAACAAACTTCAAAAAGCGAAA
TT

```
Peptide sequence:
>jgi|Dappu1|442952|dud_NCBI_GNO_4400100
MLCAFLVSASIGEVLSSSSADSNQDSVALRCADRLQQMVEAVSPAFTHALVHFDRLTDLILDATMSMKEH
LRKNAYFSELPIQRAIYWMFDSLNEAHISTLYHLKRYDDNYIRLSREIRDTEGMIKVDNDLLESLKINIS
WWAEKCEEFQKNKKNLDIKVQDAVNRVKRAEMEIEKKKTNRNGWIVAAVFTVGLATPGLIHNENKLQKAK
LKRKVYQDNANENRILLEEAEKNLKTFEAEKLITENSLKEKERNLTLLRSNKENLGKQFASLRALEEHIR
STTTFMSQFRFEVEDLKNQQDAYVLLNPLFQLVDKLSELIGSSASMVILLDLEEMVSKIRDKFGILRLQM
QN*
```

(Gray is a section that may not be included in all peptides; i.e., the gene potentially has alternate start sites)

Lipase – Upregulated in lake ecotype

Summary:

The gene LIP (lipase) is upregulated in *Daphnia pulicaria* when provided seston from Lake Murray, SC as a food source compared to when provided the lab-reared food *Ankistrodesmus falcatus*. LIP is represented on the *Daphnia* 3rd Gen microarrays by probe Dp001242 and is located at scaffold_58:367649-370285. It was previously annotated by L. Heckmann from the gene model predicted by the PASA algorithm, and we confirm the validity of the gene model Heckmann cataloged. The gene model is also supported by both EST sequences and tiling array expression.

LIP clearly shows conserved domains within the Esterase_lipase superfamily, specifically to the Pancreat_lipase_like group, according to blastp at NCBI. The best blastp hits are all to predicted pancreatic triacylglyceral lipases in arthropods including *Acyrthosiphon pisum* (8e-119), *Nasonia vitripennis* (3e-114), *Pediculus humanus* (2e-110) and *Tribolium castaneum* (1e-109). Based on the apparent link to pancreatic functions, we conclude it is likely that this gene is involved in resource assimilation. The nearest match in *Drosophila melanogaster* was to CG5966 (2e-55), a gene for which there is no experimental data but shows conserved domains of triacylglyceral lipases. The nearest match with any experimental data was to the mammal nutria (*Myocastor coypus*; Thirstrup, K et al 1995 “Cloning and expression in insect cells of two pancreatic lipases and a procolipase from *Myocastor coypus*” *Eur. J. Biochem* 227: 186-193; Thirstrup, K. et al. 1994 “evidence for a pancreatic lipase subfamily with new kinetic properties” *Biochemistry* 33: 2748-2756) and the chicken (Fendri, et al 2006 “Biochemical characterization, cloning, and molecular modelling of the chicken pancreatic lipase” *Achr. Biochem. Biophys*. 451: 149-159). Some data also exists from rodents, and apparently from arthropods.

The predicted protein is associated with GO terms for function (3824, catalytic activity; 4806, triacylyglyceraol lipase activity) and process (6629, lipid metabolism).

A tblastn to the *Daphnia pulex* genome indicates that there are six or seven related genes present in *Daphnia.* However, the probe sequence blasts only to the gene located on Scaffold 58. Thus, it appears that our observed differences in expression can be attributed to this gene.

Microarray probe sequence (Dp001242):
GATCCCACTAAACCCACCCAGTTCCTTGTCCATGGCTTCATTGATGACGGAACCGTGAGATGGATGA
AGA

Peptide sequence:
```
>jgi|Dappu1|306981|PASA_GEN_5800037
MTTASVWIISFVFLVFTGSALTGPLARESCVNESKNRVSRDFRALIKHGRDYHKDLVQKQEPSTAVLQTA
DVQQLDSKMEPITYCYGEIGCIVIDDSWYDPVHRPINQEPLPREKINTRFILHTRQRPTQDTMLYANDLD
SIRYSTFDPSKPTQFLVHGFIDDGTVRWMKRLTENLLAHGDYNVIIVNWGGGSLPMYSQATANTRVVGLE
IAYMVNTMITHFGVDPGMVHLLGHSLGAHTVSYAGERIEGLGRITGLDPAEPYFAEMPSHVRLDPTDAKF
VDAIHTDTRTILLLGYGMLEPVGHLDFYPNGGRDQPGCDPVDIALDAITEDMITGGRELAACNHLRCIEF
FIDSLVPGNTFVGYECPDNDAFHRGECTSCGADNLNCAKFGMDAALYPTRDRNYVHLLFDTDKDVPYIKY
HYGIKVNLAYPSQAEAFVYGTMRMSLYGTLGQLIDVTVADKSHKFVHGTDSFFLFVDSFNVGNIQRVELY
WKYDGTLFNPCGLFCNDHIYVRSVEISELNNYPESSRLEHTYKSCAVGADYADMKDKTWAEFYPTACV*
```

N acetyltransferase 1– downregulated in lake ecotype

Summary:

The gene NAT1 (N-acetyltransferase 1) is downregulated in *D. pulicaria* when they were provided seston from Lake Murray, SC as a food source compared to when they were provided the lab-reared food *Ankistrodesmus falcatus*. NAT1 is represented on the *Daphnia* 3rd generation microarrays by probe Dp005397, and is located at Scaffold 75: 352935-354216. It was manually curated in the *Daphnia pulex* genome sequence based on the gene model predicted by the fgenesh algorithm, and supported by evidence of expressed sequence tags and tiling array expression in wFleaBase. One apparent paralog was identified on Scaffold 81 via tblastn. The region on scaffold 81 is supported by expression data, but the sequence for Dp005397 blasts only to NAT1 on Scaffold 75.

The best match identified through blastp was to a predicted gene in *Tribolium castaneum* (E = 2e-12) that is identified as similar to CG5783. Unsurprisingly, the best match in *Drosophila melanogaster* was to CG5783 (E = 2e-09), an unnamed gene with an inferred funtion of N-actetyltransferase. The CDD showed clearly that NAT1 contains features that place it in the N-acetyltransferase superfamily. This is a large superfamily of enzymes that are involved in a variety of functions, and the role of NAT1 in responses to resources is therefore unknown. All of the key residues occur between residues 200 and 280.

Microarray probe sequence (Dp005397):
ACACTTTACATGTCCAAGCGAATGGCCCAGTCCGGCTTTCTCCCGTTCGTCAACATTGTCGTCGGCA
ACA

Peptide sequence:
```
>jgi|Dappu1|442960|dud_estExt_fgenesh1_pg.C_750067
MAGSVTLASRSDLFCLLSFLDSRLPAHCEVQMVVKAALLAANTSYQVFFLHESPSNDTYSAIVAIKKENV
ISQTQQNQKIWNLAEWSIDENALLRVYEAIDIDWKRDLVLAFNKHHNPHEKILKFLEDRHRLGEHCVYSA
GRYVLDISEAMKLQTTSLPDEVYVKSLERHHAPLIYEHWTAFKHTTTVDDIADEIDLLPSAGLFLKDNDE
LVSWIMGHAPMGMSRLFTMDGHRRKGYATLVTLYMSKRMAQSGFLPFVNIVVGNTASTKFFEGLGFRFVR
PLNAILTKPQSTDQTATSATN*
```

Neuroparsin – Downregulated in pond ecotype

Summary:

The gene Neuroparsin (previously identified and named by H. Dircksen) is downregulated in *Daphnia pulex* when they were provided seston from Lake Murray, SC as a food source compared to when they were provided the lab-reared food *Ankistrodesmus falcatus*. Neuroparsin is represented on the *Daphnia* 3rd generation microarrays by probe Dp009109, and is located at Scaffold 143:266963-269844. It was manually curated in the *Daphnia pulex* genome sequence based on the gene model predicted by the genewise algorithm, and supported by evidence of expressed sequence tags and tiling array expression in wFleaBase. We conducted our analyses using a protein sequence that includes additional residues on the 5' end relative to the cataloged gene because it did not begin with a valid start codon. No paralogs were found in the *Daphnia* genome via blastp; this appears to be a single copy gene with no related genes. The sequence of probe Dp009109 did not have blast matches anywhere in the genome other than at the Neuroparsin gene on Scaffold 143.

The CDD at NCBI showed that this gene is clearly a member of the neuroparsin superfamily. According to the CDD, Neuroparsins are produced by neurosecretory cells and have pleiotropic activities including inhibition of JH effect, stimulation of fluid reabsorption in the rectum, an increase in hemolymph lipid and trehalose, and neurotrophic effects. The strongest blastp match to the extended protein sequence was to neuroparsin 1 precursor in *Rhodnius prolixus* (E = 4e-15). The best match in *Drosophila melanogaster* was to dumpy (E = 0.28), a gene that has many effects including inhibition of serine-type endopeptidases.

Peptide sequence as curated by H. Dircksen:
```
>jgi|Dappu1|63582|e_gw1.143.36.1
CVYGIVKDYCGRDICAKGPGHRCGGKWNSLGICGEGLFCSCNRCGGCSLNTIECFNLTCI*
```

Peptide sequence that includes the first upstream, in-phase start codon:
```
>jgi|Dappu1|443019|dud_1_e_gw1.143.36.1
MQKNSRPVNSAPRCTACNFNADCPTPQN
CVYGIVKDYCGRDICAKGPGHRCGGKWNSLGICGEGLFCSCNRCGGCSLNTIECFNLTCI*
```

Microarray probe sequence (Dp009109)
ATGTAATAGGTGTGGAGGTTGTTCATTGAATACAATAGAGTGCTTTAACCTAACGTGCATATAAAAT
AA

Niemann-Pick Type C2 – Downregulated in lake ecotype

Summary:

The gene NPC2 (<u>N</u>iemann-<u>P</u>ick Type <u>C2</u>) is downregulated in *D. pulicaria* when they were provided seston from Lake Murray, SC as a food source compared to when they were provided the lab-reared food *Ankistrodesmus falcatus*. NPC2 is represented on the *Daphnia* 3rd generation microarrays by probe Dp003611, and is located at Scaffold 8:174707-175630. It was manually curated in the *Daphnia pulex* genome sequence based on the gene model predicted by the fgenesh algorithm, and supported by evidence of expressed sequence tags and tiling array expression in wFleaBase. No paralogs were found in the *Daphnia* genome via tblastn; this appears to be a single copy gene with no related genes.

The best blastp hit in *Drosophila melanogaster* was to Niemann-Pick Type C-2a (E= 3e-18), a gene where allelic variation is associated with Malpighian tubule phenotypic variants according to FlyBase. Using blastp, the best NCBI match was to an ecdysteroid-regulated protein of *Litopenaeus vannamei* (E = 5 e-20). Other matches further indicate involvement in steroid-based processes. NCBI's conserved domain search indicates it is part of the ML

superfamily, which have diverse functions all centered on interactions with lipids. Mutation of the human homolog is associated with Niemann-Pick type C disease, which leads to accumulation of LDL cholesterol in lysosomes.

Microarray probe sequence (Dp003611):
TCGGTGAGTGCTAAGGCCATCTGGAACCTCCGTGACGATAACGGCAATCCTCTTGTTTGCTTCGAAG
TTA

Peptide sequence:
>jgi|Dappu1|230477|estExt_fgenesh1_kg.C_80003
MFKLAIAATCALLFVAVSATPYRDCGSTATLTAVRVPGCDVLPCIVYRGTNVSVQYDFVAVDSTNTLDSD
VKGVIGGVTLPWPGQFPPACEDAIVGDCPVTAGESMTMSTILVLSPSFPSVSAKAIWNLRDDNGNPLVCF
EVTVKLL*

Peritrophic Matrix Protein 1 -- upregulated in lake ecotype
Summary:

The gene PTM1 (Peritrophic matrix protein 1) is upregulated in *Daphnia pulicaria* when they were provided seston from Lake Murray, SC as a food source compared to when they were provided the lab-reared food *Ankistrodesmust falcatus*. PTM1 is represented on the *Daphnia* 3rd Gen microarrays by probe Dp001627, and is located at Scaffold 13: 916646-917000. It was manually curated in the *Daphnia pulex* genome sequence based on the gene model predicted by the NCBI Gnomon algorithm, and supported by expression evidence from ESTs and tiling array expression in wFleaBase. Although a tblastn identified no definite paralogs, some similarity was found to two genes (on scaffolds 5 and 220) that may also be members of the Peritrophin/Obstructor family of genes. tBlastn did not recover similarity to PTM2, on scaffold 66, suggesting that they represent different classes of peritrophic matrix proteins.

PTM1 is strongly suggested to be associated with the peritrophic matrix (= peritrophic membrane), which forms the inner lining of the gut. Over a span of ~30 amino acids, the protein contains a Peritrophin-A domain that places it in the CBM_14 Superfamily. The pertitrophin-A domain is found in chitin-binding proteins (it is a type 2 chitin-binding domain), particularly peritrophic matrix proteins of insects and animal chitinases. It is an extracellular domain that contains six conserved cysteins that probably form three disulphide bridges. Manual examination of the *Daphnia* sequence shows all six cysteins occupying the characteristic spacing (highlighted in red below).

Microarray probe sequence (Dp001627)
TGTAGGAACTTGTCCGTATCCGGATGGACCGAATCCAGTTTACTTAACAGACTCTGTTAGATGCGAT
GTG

>jgi|Dappu1|315109|NCBI_GNO_1300171
MKSTSLPLVFLAVLIAVSLPGSTAVPVGTCPSVDGPNPVYLTDSVRCDVFYECSNGYANEKVCPNNPYAN
PPANLHWNKALSVCDFPQNANCLL*

Peritrophic Matrix Protein 2 – upregulated in both ecotypes
Summary:

The gene PTM2 (Peritrophic matrix protein 2) is upregulated in both ecotypes when they were provided seston from Lake Murray, SC as a food source compared to when they were provided the lab-reared food *Ankistrodesmust falcatus*. PTM2 is represented on the *Daphnia* 3rd Gen microarrays by probe Dp008924, and is located at 66:473834-475076. It was manually

curated in the *Daphnia pulex* genome sequence based on the gene model predicted by the PASA algorithm, and supported by expression evidence from ESTs and tiling array expression in wFleaBase. tBlastn suggests there may be a paralog on scaffold 53 (E = 2e-31) that may also be in the Peritrophin/Obstructor family of genes. tBlastn did recover similarity to PTM1 (E = 2e-08), on scaffold 13, despite the reverse not showing a match.

All functional inferences are identical to those for PTM1.

Microarray probe sequence (Dp008924)
AGCGCTTATTTCGGGAAATCTTCTGTTTCCGGGGGCTAAATACATTTTCTAGTTAAGGAAAGAAATAA
GG

```
Peptide sequence:
>jgi|Dappu1|442947|dud_PASA_GEN_6600009
MFLRCLLITLVTAAFLKRMQATEMDSQETDGRVSVCPEDGVYPNYYNCSTFITCSNGIQYLMACPEGLIW
NVDTSECDWPNNTECVTYPAIRIDYWVVERLFREIFCFRRLNTFSMQYHSIFYLIYSIIIFNPINGIRCD
*
```

Punch – downregulated in pond ecotype.

Summary:

The gene Punch is downregulated in *Daphnia pulex* (the pond ecotype) when they were provided seston from Lake Murray, SC as a food source compared to when they were provided the lab-reared food *Ankistrodesmus falcatus*. Punch is represented on the *Daphnia* 3rd generation microarrays by probe Dp007040, and is located at Scaffold 7:2239290-2243701. It was manually curated in the *Daphnia pulex* genome sequence based on the gene model predicted by the PASA algorithm, and supported by evidence of expressed sequence tags and tiling array expression in wFleaBase. Two potential paralogs were found in the *Daphnia* genome via tblastn on Scaffolds 11105 and 12246; both are partials with no evidence of expression.

Conserved domain analysis at NCBI clearly shows that Punch is a GTP cyclohydrolase I, a group of enzymes that catalyzes the conversion of GTP into dihydroneopterin triphosphate. Blastp showed that the best match in *D. melanogaster* was Punch (E = 2e-79), and overall (E = 7e-81) was was to GTP cyclohydrolase in the hemichordate *Saccoglossus kowalevskii*. In *Drosophila*, there is experimental evidence that it is involved with several biological processes, including preblastoderm mitotic cell cycle; cuticle pigmentation; pteridine biosynthetic process; embryonic pattern specification; compound eye pigmentation; larval chitin-based cuticle development.

Microarray probe sequence (Dp007040):
TTCAGAAGATCAACGCCAAGACAGTCACTTCCGCCATGTTGGGCGGTTTCCGTGATGACTCCAAGAC
ACG

```
Peptide sequence:
>jgi|Dappu1|442958|dud_PASA_GEN_0700094
MEAFKGSKPVAGAAVTNGDDPECKSITNGLRTIDVDNLRSSPRLNREDMLPSMVESYRSLLSDVGEDPCR
QGLLKTPERAAKAFLFFTKGYEQTLEEVLNDAIFDENHDEMVVVKDIEMFSMCEHHLVPFIGKVSIGYLP
NGKVLGLSKLARIVEIYSRRLQVQERLTKQIALAVCEAVTPHGVGVVVEATHMCMVMRGVQKINAKTVTS
AMLGGFRDDSKTREEFLTFVRTP*
```

Regulated by Field Seston 1 – Downregulated in both ecotypes

Summary:

The gene RFS1 (Regulated in Field Seston 1) is downregulated in both *Daphnia pulex* and *D. pulicaria* when they were provided seston from Lake Murray, SC as a food source compared to when they were provided the lab-reared food *Ankistrodesmus falcatus*. RFS1 is represented on the *Daphnia* 3rd generation microarrays by probe Dp000454, and is located at Scaffold 5: 51058-51958. It was manually curated in the *Daphnia pulex* genome sequence based on the gene model predicted by the PASA algorithm, and supported by evidence of expressed sequence tags and tiling array expression in wFleaBase. No paralogs were found in the *Daphnia* genome via tblastn; this appears to be a single copy gene with no related genes.

The function of RFS1 is unknown, and no homologs were identified in Arthropods. Using blastp, very weak hits (e-values of -2 or higher) were found in mammals, hemichordates and *Caenorhabditis*, and none of the homologs had any functional information. No conserved domains were identified via NCBI. The best match (0.002) was to a hypothetical protein (LOC100374271) in the hemichordate *Saccoglossus kowalevskii*. According to WormBase, RNAi knockouts of the apparent homolog in *C. elegans* (WBGene00019518) yielded no observed phenotype.

Microarray probe sequence (Dp000454):
ATGACAGAGTATTCAGCCATCGTGATGTACTCTCTACCAATGGACACGACTGGTTCGGGTTTGTGGG
GAG

Peptide sequence:
>jgi|Dappu1|442953|dud_PASA_GEN_0500139
METRVKVFGFFDILLGITMIMTEYSAIVMYSLPMDTTGSGLWGGMFVMVTGVVIIKRHNIMVLVFSILSA
MSGIIMICLYIWSFTLYGDMISSGYTCGATLYMGHSICSRIALDSLFLIYGVAALCMNTILIHDAKWIEP
HKKQNEAPALTNIPVVVTITPAAINGHNLSS*

Regulated by Field Seston – downregulated in lake ecotype

Summary:

The gene RFS2 (Regulated in Field Seston 2) is downregulated in *Daphnia pulicaria* when they were provided seston from Lake Murray, SC as a food source compared to when they were provided the lab-reared food *Ankistrodesmus falcatus*. RFS2 is represented on the *Daphnia* 3rd generation microarrays by probe Dp004320, and is located at Scaffold 29:81845-83186. It was manually curated in the *Daphnia pulex* genome sequence based on the gene model predicted by the PASA algorithm, and supported by evidence of expressed sequence tags and tiling array expression in wFleaBase. No paralogs were found in the *Daphnia* genome via blastp; this appears to be a single copy gene with no related genes.

The best blast match was to a hypothetical protein in *Myxococcus xanthus* (E = 5e-17). Other matches better than e-2 were also to hypothetical proteins with no functional information. A blastp specifically for *Drosophila melanogaster* showed weak similarity (E = 5.7) to the gene *kuzbanian*, a gene involved in phagocytosis in the innate immune response. Though weak, matching residues were spread across the length of the genes. No information was available on whether matching residues were associated with functionally significant sites in *kuzbanian*. NCBI's conserved domain database identified no functional regions either.

Microarray probe sequence (Dp004320):
CGTATCTTTGCCACATTTCAGAGGCGAAAATTTGTGCGATTTGAGAGGCTGTGAAGGCGGGAATCTT
CAA

>jgi|Dappu1|304421|PASA_GEN_2900013
MFVKWALLFSFALVFFNVSECVKDEVHELHDLVVQYAPRLRFDSKSGDTLGQCLPSSAEDYFRLRQNGFT
GRVCNMDYISILDGRIPAYYEADECENNNMIFSYWFFYGYKDDCPMLPGDPGDDVNWGRYVVKVLNGSQV
DRVIFYQHEGWYTRNPGRYEVFESTHPISYVGKLRQGTYHDDGGSGTCCYFEDYRNPGSLDKHMDSWRNL
VWLRNNGNTTPEWMTNNDPYYWNGVPLPHFRGENLCDLRGCEGGNLQTCGTCGCHKSDVADDERP*

Truncated Monooxygenase – downregulated in lake ecotype

Summary:

The gene tMOX (truncated Monooxygenase) is downregulated in *D. pulicaria* when they were provided seston from Lake Murray, SC as a food source compared to when they were provided the lab-reared food *Ankistrodesmus falcatus*. TMOX is represented on the *Daphnia* 3rd generation microarrays by probe Dp006883, and is located at Scaffold 5: 51058-51958. It was manually curated in the *Daphnia pulex* genome sequence based on the gene model predicted by the PASA algorithm, and supported by evidence of expressed sequence tags and tiling array expression in wFleaBase. The gene has a premature stop codon that would lead to a truncated, potentially non-functional, protein. EST sequences in wFleabase run across the premature stop, and suggest that the premature stop may be an error in the genomic sequence. No paralogs were found in the *Daphnia* genome via tblastn with the entire, untruncated protein sequence; this appears to be a single copy gene with no related genes. The sequence for probe Dp006883 shows a blast match only to TMOX on scaffold 16, and is located after the premature stop codon.

Blastp found only weak matches, the best being to a hypothetical protein in *Branchiostoma floridae* (E = 0.001) and "similar to CG5235" in *Saccoglossus kowalevskii* (E= 0.009). CG5235 is a dopamine beta monooxygenase in *Drosophila melanogaster*, but the best blastp hit in *D. melanogaster* is actually to olf314 (E = 0.089), a different dopamine beta-monooxygenase. There were no matches in the NCBI conserved domain database.

Microarray probe sequence (Dp006883)
CCCATGATGATTGGATGGGCCTGGTCGACCGGCATCTTCAACAACGGATTTCTGACCCCCATCGAG
AAAT

Truncated peptide sequence:
>jgi|Dappu1|302152|PASA_GEN_1600127
MMKFLMIAASLAAICSALPSRLGQQTRQSGRELESMMLNNTKELDPEGSFRLDWDVVYEDPSNPLLVLEM
RVATTGWFSLRFTSADLTLGDYFYGA*

Untruncated peptide sequence:
>jgi|Dappu1|442955|dud_PASA_asmbl_4703
MMKFLMIAASLAAICSALPSRLGQQTRQSGRELESMMLNNTKELDPEGSFRLDWDVVYEDPSNPLLVLEM
RVATTGWFSLRFTSADLTLGDYFYGA*DASKPSNSFFLDKHCALVDGLGCETAEGPRDDFRNDFQLLSFV
FGTDYTVMRIARQVDTGNNLQDVVISPGPMMIGWAWSTGIFNNFGFLTPIEKFGFQTVALIAAE*

UDP-glucuronyltransferase 1 – downregulated in pond ecotype

Summary:

The gene UGT1 (UDP-glucuronyltransferase 1) is downregulated in *Daphnia pulex* when they were provided seston from Lake Murray, SC as a food source compared to when they were provided the lab-reared food *Ankistrodesmus falcatus*. UGT1 is represented on the *Daphnia* 3rd generation microarrays by probe Dp004865, and is located at Scaffold 135:122472-124405. It was manually curated in the *Daphnia pulex* genome sequence based on the gene model predicted by the Gnomon algorithm, and supported by evidence of expressed sequence tags and tiling array

expression in wFleaBase. Many paralogs were found in the *Daphnia* genome via blastp, which is unsurprising because the UGTs are a large family of genes in other arthropods. However, the sequence for probe Dp004865 matched only the gene on Scaffold 135. It was named UGT1 because we could not confidently assign homology to a particular member of the UGT family.

The best blastp match in *Drosophila melanogaster* was to CG30438 (E = 3e-67), an apparent member of the UGT family, whereas the best match to a named gene was to Ugt86Da (E = 9e-63). In a blastp to the entire NCBI database, the best matches were to *Tribolium castaneum* genes identifed as similar to the afore-mentioned genes in *D. melanogaster*. The CDD search at NCBI showed that the gene is clearly a member of the glycosyltransferases, in particular showing the features (between residues 345 – 430) of UDP-dependent glucuronyltransferases, which are involved in carbohydrate transport and metabolism.

Microarray probe sequence (Dp004865)
TGGAATGAGATCGAAGACAAGCTGCTCCACCGCACCATCCGTCAGCTGATTTACCAAGATTCGTACG
TGG

Peptide sequence:
>jgi|Dappu1|330188|NCBI_GNO_13500025
MSFLPARLSVFFVLIGLASCHNILVFMPFGSHSHKATLIPLIQGLLERNHSVTFITNQESTDLRYHKMMY
NLDEVVVPNLNYSMLDPESAEQNFFEIAAKQSIRSQLKIFSHLSGQVNRVLDQTYSDAGVQSVLRHGQFD
LLLLSQVVSYAGYPMAWHFQCPFILSSPNVLMTDSAYLLGDSEHTEYVPFFLMALTDQMNLVQRVINTVV
THLLNFYQDMFVFPRLQPAIEKYFPGAPSLIEMKANITAAFANTHPAFSYPRAYPPGVVELGGIHCRPAK
PLPHRLEQFVAESGSAGFIVFGVGSIIPMDEMPREMLDVFIRVFSRLPQRVVWQWRGFNKPANLSDNILL
VDWLPQQDLLGHEKCRLFLTHGGLLSTQEAIYHGVPVLGLPFISDQLLNMDKAVRDGYALQLRWNEIQDK
LLHRTIHELIYQNSYVENVRRRQSLLLDQSESPLERGLYWAEYIARHGGAPHLQLGSRHLNRFQRSLVDV
YLILAAIACLLIFATWRCLGRFEFVARLTKLDFF*
